# Old Things New View: Ascorbic Acid Protects the Brain in Neurodegenerative Disorders

**DOI:** 10.3390/ijms161226095

**Published:** 2015-11-27

**Authors:** Adriana Covarrubias-Pinto, Aníbal Ignacio Acuña, Felipe Andrés Beltrán, Leandro Torres-Díaz, Maite Aintzane Castro

**Affiliations:** 1 Instituto de Bioquímica y Microbiología, Facultad de Ciencias, Universidad Austral de Chile, Valdivia 5090000, Chile; adriana.covarrubias@postgrado.uach.cl (A.C.-P.); aacuna.sanmartin@alumnos.uach.cl (A.I.A.); felipe.beltran@alumnos.uach.cl (F.A.B.); leandro.torres@postgrado.uach.cl (L.T-D.); 2 Center for Interdisciplinary Studies on the Nervous system (CISNe), Universidad Austral de Chile, Casilla 547, Valdivia 5090000, Chile

**Keywords:** oxidative stress, brain energy metabolism

## Abstract

Ascorbic acid is a key antioxidant of the Central Nervous System (CNS). Under brain activity, ascorbic acid is released from glial reservoirs to the synaptic cleft, where it is taken up by neurons. In neurons, ascorbic acid scavenges reactive oxygen species (ROS) generated during synaptic activity and neuronal metabolism where it is then oxidized to dehydroascorbic acid and released into the extracellular space, where it can be recycled by astrocytes. Other intrinsic properties of ascorbic acid, beyond acting as an antioxidant, are important in its role as a key molecule of the CNS. Ascorbic acid can switch neuronal metabolism from glucose consumption to uptake and use of lactate as a metabolic substrate to sustain synaptic activity. Multiple evidence links oxidative stress with neurodegeneration, positioning redox imbalance and ROS as a cause of neurodegeneration. In this review, we focus on ascorbic acid homeostasis, its functions, how it is used by neurons and recycled to ensure antioxidant supply during synaptic activity and how this antioxidant is dysregulated in neurodegenerative disorders.

## 1. Introduction

Ascorbic acid, the reduced form of vitamin C, is an essential metabolite for a variety of organisms. It is present in multiple fruits and vegetables [1] and is also synthesized from glucose in the liver of many mammalian species, allowing the maintenance of physiological levels. However, higher primates, including humans, lack the functional enzyme for the final step of synthesis, rendering them dependent on exogenous sources of ascorbic acid [2,3]. This dietary dependence led to its categorization as a vitamin upon the discovery that its deficiency caused scurvy. Ascorbic acid is an important antioxidant with multiple cellular functions. It plays a role in detoxification processes [4,5,6], participates as an enzymatic cofactor [7,8,9] modulates synaptic activity and neuronal metabolism [5,10,11,12], among other functions.

Ascorbic acid is concentrated in the brain [13]. The brain is responsible for the 25% of total body glucose utilization [14,15]. Such elevated activity correlates with a high oxidative metabolism and therefore the brain is dependent on antioxidants for protection against pathological conditions [5,16]. Redox imbalance and oxidative stress are observed during aging [17,18,19] and in neurodegenerative disorders such as Alzheimer’s disease (AD), Parkinson’s disease (PD), Huntington’s disease (HD), Amyotrophic lateral sclerosis (ALS), among others [5,20,21,22,23,24].

Imbalance of ascorbic acid homeostasis has also been demonstrated in neurodegenerative disorders [22,23,25,26]. During the neurodegeneration process a clear link exists between ascorbic acid deficiency and oxidative-induced neuronal death. Therefore, emphasis should be placed on novel target discovery to prevent or fight oxidative stress, especially when designing molecules to ensure the correct supply of antioxidants, such as ascorbic acid, and developing new strategies to alleviate neurodegeneration progression [27,28].

In this review, we will summarize the ascorbic acid functions in the Central Nervous System (CNS), mechanisms for ascorbic acid uptake and recycling, the relationship between redox imbalance and oxidative stress, and ascorbic acid homeostasis failure observed in several neurodegenerative disorders.

## 2. General Aspects of Ascorbic Acid

Ascorbic acid is involved in the first line of antioxidant defence, protecting lipid membranes and proteins from oxidative damage [6,29]. Ascorbic acid is synthesized in the liver of most mammals through the formation of d-glucuronic acid and l-gulono-γ-lactone. Humans, other primates, and guinea pigs do not express the functional enzyme l-gulono-γ-lactone oxidase and are therefore unable to synthesize ascorbic acid [30,31]. Thus, humans require a constant supply of ascorbic acid from fruit and vegetables. In this regard, mechanisms for ascorbic acid transport and recycling are important for ensuring distribution of this molecule in the brain [1,32,33]. The optimal dose of ascorbic acid to fully saturate plasma and tissues in healthy adults is 500 mg [34]. Which far exceeds the recommended dosis (60 mg) to prevent deficiency diseases [35] and promote general health [36]. At doses greater than 500 mg urinary excretion of ascorbic acid is increased without changes of plasma levels [37,38].

### 2.1. Ascorbic Acid Uptake and Distribution in the CNS

Specific transporter systems actively driven by the sodium gradient are responsible for ascorbic acid uptake. Members of the transporter family SLC23, the Sodium dependent Vitamin C transporters 1 and 2 (SVCT1 and SVCT2), are expressed in multiple cell types [39,40,41]. In regards to vitamin C transporter expression in brain, SVCT2 is exclusively expressed in neurons [39,41,42] hypothalamic glial cells [43] and epithelial cells from the choroid plexus [41]. The apical SVCT1 transporter participates in intestinal absorption, in the lumen of intestinal epithelium, and renal reabsorption of ascorbic acid [44,45]. Once ascorbic acid enters the bloodstream it is distributed to different organs. In the CNS, it is not fully understood how ascorbic acid is transported through the blood- cerebrospinal fluid (CSF) barrier [13] and blood-brain barrier (BBB) in endothelial cells of brain blood vessels [46] (Lam and Daniel, 1986). SVCT2 is expressed in the basolateral membrane of choroid plexus epithelia [47] therefore ascorbic acid is taken up from the blood into the intracellular space of epithelial cells. However, it is still unknown how ascorbic acid is released from choroid plexus cells to the CSF. SVCT2 expression at the BBB is not clear [48]. The entry of ascorbic acid to the brain via the BBB has been proposed to involve the SLC2A family of hexose transporters (GLUT) which are able to translocate dehydroascorbic acid; the oxidized form of vitamin C. It should be considered that under physiological conditions vitamin C is present in the body mainly in the reduced form, ascorbic acid [6,49]. Thus, local oxidation of ascorbic acid to dehydroascorbic acid, uptake of the oxidised form by endothelial cells through GLUTs and intracellular reduction back to ascorbic acid should be necessary [8,50,51,52,53].

The brain exhibits one of the highest ascorbic acid concentrations in the body, with decreases of less than 2% per day [54]. Studies using mice have shown that the brain preferentially retains ascorbic acid at the expense of other tissues [5]. In whole brain, 1 to 2 mM of ascorbic acid has been detected while intracellular neuronal concentrations are much higher, reaching up to 10 mM [54,55]. Cortex, nucleus accumbens, hypothalamus, hippocampus, choroid plexus and cerebrospinal fluid exhibit high concentrations of ascorbic acid [13,31,56]. In rat brain, ascorbic acid is present in CSF at a concentration of 500 μM [57]. Since ascorbic acid is found in cerebral extracellular space at a concentration of 200–400 μM [49,57,58], while blood levels are around 60 μM [13], it highlights evidence of an active role of ascorbic acid uptake in the brain. Hence, regulation of ascorbic acid uptake and recycling are vital for its homeostasis.

#### 2.1.1. Ascorbic Acid Homeostasis and Synaptic Activity

In mammalian tissues, CNS neurons are of particular interest because they contain high ascorbic acid concentrations, as mentioned above. Ascorbic acid is accumulated in neurons as a result of SVCT2 expression, the specific transporter for ascorbic acid [39,41,42]. Neurons are sensitive to ascorbic acid deficiency since they have an oxidative metabolism rate 10-fold higher than glial cells [16,59]. It has been suggested that ascorbic acid has a neuroprotective role due to the existence of homeostatic mechanisms which maintain high concentrations of ascorbic acid in CSF and neurons [4,5,16].

Ascorbic acid concentration in the brain changes according to neuronal activity. Thus, in response to brain activity, extracellular ascorbic acid concentrations are increased [60,61,62,63]. Accordingly, ascorbic acid efflux induced by synaptic activity from glial intracellular reservoirs can sustain the antioxidant requirements of active neurons and their high oxidative metabolism. A mechanism for neuronal ascorbic acid uptake-modulation was described by Acuña and colleagues, where an increase in extracellular ascorbic acid concentration (that occurs during synaptic activity) increases cell surface expression of the SVCT2 transporter, thus increasing ascorbic acid uptake [26].

**Figure 1:**
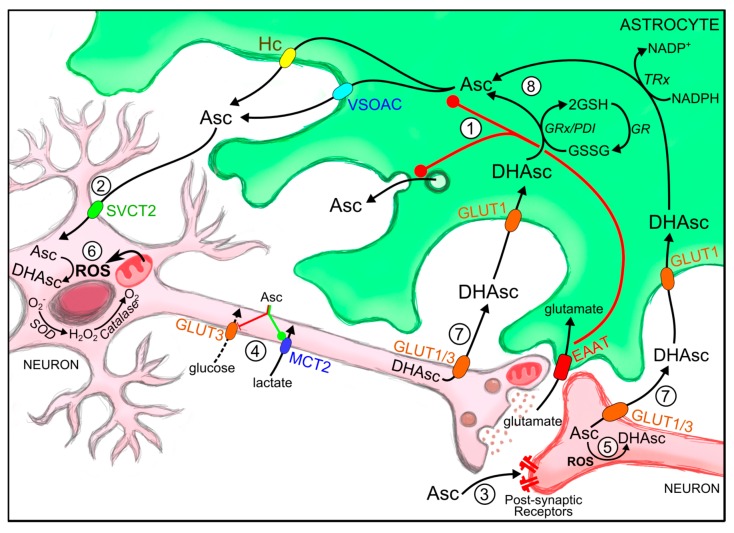
Roles of ascorbic acid during synaptic activity and astrocyte-mediated recycling. (1) During synaptic activity glutamate released into the synaptic space is taken up by astrocytes, where it stimulates ascorbic acid release from these cells through different hypothesized mechanisms (after astrocyte swelling by VSOAC induced via GLAST glutamate-aspartate transporter; Connexin-hemichannels; or through exocytosis of secretion vesicles); (2) Astrocyte-released ascorbic acid is taken up by neurons through SVCT2; (3) It participates as a neuromodulator (glutamatergic and GABA-ergic neurotransmission); (4) Regulates neuronal metabolic substrate preference via specific GLUT3 inhibition (metabolic switch). Reactive oxidant species (ROS) produced during synaptic activity (5) and neuronal metabolism (6) oxidize ascorbic acid to DHAsc; (7) DHAsc is released from neurons and is taken up by astrocytes through glucose transporters (GLUTs); (8) Astrocytes can reduce oxidized ascorbic acid via direct reaction with glutathione o via glutathione-dependent reductases such as glutaredoxin and protein disulfide isomerase. AMPAR, (2-amino-3-(3-hydroxy-5-methyl-isoxazol-4-yl) propanoic acid receptor; Asc, ascorbic acid; DHA, dehydroascorbic acid, oxidized ascorbic acid; EAAT, excitatory amino acid transporter; GABA (γ amino butyric acid); GLAST, (glutamate transporter in astrocytes); GLUT3, glucose transporter isoform 3; GSH, reduced gluthatione; GSSG, oxidized gluthatione; MCT, monocarboxylate transporter; ROS, reactive oxygen species; SVCT2, sodium-vitamin C transporter isoform 2; VSOAC, Volume-sensitive organic osmolyte-anion channel.

Ascorbic acid is a potent reductant. When it carries out its antioxidant activity it is oxidized [64]. However, the reduction of its oxidised form is not a spontaneous event. It is an enzymatic reaction, which may be a glutathione-dependent [7,8,9,65,66] or –independent [67,68,69] reaction (Figure 1). The mechanisms for ascorbic acid efflux and recycling carried out by astrocytes will be mentioned below.

#### 2.1.2. Mechanisms for Astrocytic Ascorbic Acid Recycling and Efflux

Neurons exhibit high rates of oxidative metabolism. Oxidative processes generate free radicals which are scavenged by ascorbic acid. Neuronal cells express dehydroascorbic reductase which could be involved in ascorbic acid regeneration from its oxidized form; however, this enzyme is only expressed in pyramidal and Purkinje neurons. Hence, astrocytes take up and recycle ascorbic acid from the extracellular space participating in a coupling mechanism between astrocytes and neurons, where oxidative processes are mainly compartmentalized in neurons leading to the oxidation of ascorbic acid to dehydroascorbic acid. Facilitative glucose transporters (GLUTs) are responsible for dehydroascorbic acid uptake by CNS cells [4,70,71,72]. Neurons can release dehydroascorbic acid through GLUT3 or GLUT1 and then it is taken up by astrocytes where transport is restricted to a single isoform, GLUT1 (Figure 1). Moreover, studies show that primary cultures of astrocytes exclusively take up dehydroascorbic acid via a Na^+^-independent manner and are unable to transport ascorbic acid.

Glutathione (GSH, c-l-glutamyl-l-cysteinylglycine), vitamin E, and ascorbic acid are highly concentrated in astrocytes. They also express ROS-detoxifying enzymes such as GSH S-transferase, GSH peroxidase, and catalase [16,73,74,75]. Therefore astrocytes are resistant to oxidative damage [76,77]. After an oxidative insult, astrocytes undergo several processes which include expression of antioxidant enzymes [77,78] and glucose oxidation through the pentose phosphate pathway, producing reducing agents for antioxidant regeneration [79,80,81,82] such as GSH synthesis for ascorbic acid recycling [77,78]. Oxidized ascorbic acid is thus reduced in the glial intracellular milieu and accumulated within astrocytes (Figure 1, [83]) and then the ascorbic acid could be released to the extracellular space in response to synaptic activity where it contributes to neuronal antioxidant defence [26,61,62,84].

Since dehydroascorbic acid is a toxic molecule for the brain [85], astrocytes must rapidly reduce it in order to prevent dehydroascorbic acid retrotransport back to the extracellular space. Several pathways have been proposed for dehydroascorbic acid recycling in glial cells. Enzymatic reduction of dehydroascorbic acid has been described where GSH acts as a cofactor [86,87]. This could be conducted by GSH-dependent enzymes such as glutaredoxin, protein disulfide isomerase [88,89,90], and GSH S-transferase ω [65,91,92]. Other mechanisms of intracellular dehydroascorbic acid reduction implicate NADPH-dependent reductases such as 3α-hydroxysteroid dehydrogenase [93] and thioredoxin reductase [94,95,96]. Since astrocytes express four times more GSH than neurons they exhibit a higher capacity to reduce ascorbic acid [54]. Indeed, incubation with 0.2 mM dehydroascorbic acid can decrease the intracellular concentration of GSH in cultured astrocytes [97].

Astrocytes are more efficient at scavenging ROS than neurons. Defence mechanisms against oxidative stress are essential for neuronal viability [16,73,98,99,100]. Astrocytes attenuate neuronal death caused by hydrogen peroxide when they are co-cultured with striatal neurons [74]. Several studies have shown that cultured neurons in the presence of astrocytes are more resistant to pro-oxidant agents such as hydrogen peroxide [101,102], nitric oxide [98,103], superoxide anion combined with nitric oxide, [104] or iron [98,104]. This glial neuroprotection has been detected at an astrocyte-neuron ratio of 1:20 [16].

A correlation exists between brain activity and extracellular ascorbic acid concentration [60,61,62,84]. Since astrocytes accumulate ascorbic acid they are suitable candidates for supplying this antioxidant in response to synaptic activity. In fact, neurotransmitter-stimulated release of ascorbic acid has been observed in cultured astrocytes (Figure 1, [105]). For example, glutamate released during synaptic activity, is cleared from the synaptic cleft by astrocytes through the glutamate transporter GLT1, to prevent excitotoxicity-induced neuronal death [106,107]. Furthermore, ascorbic acid seems to antagonize the negative effects of glutamate [31,108,109,110]. Glutamate-induced cellular swelling has been proposed as a mechanism for astrocytic ascorbic acid release [61,84,111,112], in fact, this release is observed through cell swelling alone [113]. Ascorbic acid release induced by hypotonic conditions was impeded by anion-transport inhibitors (4,4′-diisothiocyanostilbene-2,2′-disulfonic acid; DIDS and 4,4′-dinitrostildene-2,2′-disulfonic acid; DNDS) suggesting anion-regulated release of ascorbic acid mediated by VSOAC (volume-sensitive organic osmolyte-anion channel, [97,113,114]). This should be considered in cases of ischemia where ion transport mechanisms regulating cell volume are reduced and could thus induce astrocyte swelling [115]. Ascorbic acid may also be released from astrocytes via other mechanisms. Studies have suggested ascorbic acid release through connexin hemichannels using Conexin 26 and 32 loaded liposomes [116,117,118]. Finally, ascorbic acid release through secretory vesicles has been described in parathyroid glands and adrenomedular vesicles after adrenergic and nicotinic stimulation [119].

## 3. Roles of Ascorbic Acid at the CNS

The main role of intracellular ascorbic acid in the CNS is to provide antioxidant protection against oxidative damage [68]. However it also functions as an enzymatic co-factor, participating in collagen [120,121,122,123], carnitine [124], tyrosine and peptidic hormone biosynthesis [125,126]. Severe dietary deficiency of ascorbic acid can cause health disorders such as scurvy syndrome [127,128,129]. Ascorbic acid deficiency has a negative impact on brain function particularly during development [130]. A high growth rate in a developing brain is responsible for an increased cellular metabolism. This together with an immature antioxidant system promotes redox imbalance [131]. Thus, levels of ascorbic acid in brain must be increased during early life [132]. Ascorbic acid deficiency during guinea pig pregnancy leads to persistent impairment of postnatal hippocampal development supporting the idea that maternal ascorbic acid deficiency exhibits severe consequences for the offspring [133]. In fact, the absence of ascorbic acid in the brain has been shown to be detrimental to newborn SVCT2 (−/−) mice survival [5,134].

Ascorbic acid is considered an important neuroprotective agent [124,135,136] since it is a potent reducing agent, scavenging ROS production and sustaining superoxide dismutase and catalase activities [137]. It also protects neurons against glutamate excitotoxicity, which is associated with neurodegenerative processes [138,139,140,141]. However, ascorbic acid functions in the brain are broader than those based on its antioxidant properties (Figure 1).

### 3.1. Ascorbic Acid as a Neuromodulator of Synaptic Activity

Ascorbic acid affects synaptic neurotransmission by preventing neurotransmitter binding to receptors [142,143,144], by modulating their release and reuptake [145], and also acting as a cofactor in neurotransmitter synthesis [146,147]. Therefore, ascorbic acid is considered a nerudomodulator of neural transmission [5]. It could modulate synaptic receptors by reducing aminoacidic residues [19,148] or by scavenging reactive oxygen species generated through neurotransmitter receptor activation [143,144]. Also, ascorbic acid participates in the conversion of dopamine to norepinephrine [5,54] and it is essential for norephinephrine and acetylcholine release from synaptic vesicles [149]. Studies show that ascorbic acid modulates the activity of glutamate receptors, voltage-activated ionic channels, such as the neuronal T-type calcium channel (Ca^+2^ (v) 3.2 T-channel) and the voltage-dependent K^+^ currents [144,150,151], and GABA receptors [152]. On the other hand, myelin formation in Schwann cells could be stimulated by ascorbic acid [153].

### 3.2. Ascorbic Acid and the Ascorbic Acid Metabolic Switch

Ascorbic acid can modulate neuronal metabolism switching the energy substrate preference from glucose to lactate during synaptic activity. The neuron-glia metabolic coupling model has gained force over the past decade, especially when tripartite synapse was described as a functional and structural unit, comprised of pre and post-synaptic terminals and glial processes surrounding the synaptic cleft [154].

The uptake of glutamate is able to stimulate glucose uptake, glycolysis and lactate release in astrocytes [155,156]. The astrocyte-neuron lactate shuttle (ANLS) hypothesis describes a functional mechanism of synaptic activity where lactate release from astrocytes is stimulated by glutamate uptake, and is then taken up by neurons to support their energetic needs. Neurons can take up released lactate through the monocarboxylate transporter 2 MCT2 [157,158]. This lactate flux is possible due to differential expression of monocarboxylate transporters and lactate dehydrogenase isoenzymes in neuronal and astroglial cells [159,160,161,162]. However, lactate transport and utilization by neurons is strongly debated since neurons are suitable for using glucose as their metabolic substrate [163,164,165,166,167,168,169,170]. In this context, the ascorbic acid metabolic switch proposes a mechanism responsible for the inhibition of neuronal glucose utilization during synaptic activity.

During the ascorbic acid metabolic switch, ascorbic acid released from glial cells is taken up by neurons where it inhibits glucose transport and utilization. This allows lactate uptake and metabolization as the primary energy source in neurons [10,11]. We have demonstrated neuronal inhibition of glucose usage by intracellular ascorbic acid through a mechanism involving GLUT3 [171]. Evidence of ascorbic acid recycling in the brain supported that idea [83] and it is independent of the antioxidant properties of ascorbic acid [172]. At the same time, intracellular ascorbic acid is capable of stimulating lactate uptake in neurons in a GLUT3-dependent manner [11].

## 4. High Metabolism and ROS Production by Neurons

### 4.1. High Neuronal Metabolism during Synaptic Activity

The brain is in charge of processing and regulating several physiological functions of the body, explaining its elevated energetic demands. In fact, even at rest the CNS accounts for up to 25% of oxygen consumption in adults [14,173]. Most of the energy utilized can be attributed to neuron and synapse related processes. Restoration of the membrane gradient after depolarization is the main energy demand of neurons. However, neurotransmitter recycling, dendritic and axonal transport, and intracellular signaling also account for a significant part of energy consumption [14]. Given that over 80% of neurons are excitatory and over 90% of synapses release glutamate, the major player in neuroenergetics is glutamatergic activity [174,175,176].

The CNS relies on glucose metabolism to sustain the energetic cost of synaptic activity. This molecule is a good source of potential energy, making it an efficient metabolic fuel and whose metabolism correlates with brain function [60,61,62,84,177,178]. There is a continuous glucose flow across the BBB through the glucose transporter GLUT1 [50,179,180,181], which is then taken up by neurons through GLUT3 and astrocytes through GLUT1. Glycolytic activity breaks down glucose to pyruvate in all brain cells, where the enzymes hexokinase, phosphofructokinase 1, and to a lesser extent pyruvate kinase are the key control sites [182,183]. Lactate dehydrogenase is responsible for the conversion of pyruvate to lactate in glial cells and according to the previously mentioned ANLS hypothesis lactate is released to the extracellular space and taken up by neurons [155,158,184]. While glycolysis has a net yield of 2 ATP molecules, if glucose is subsequently oxidized through the tricarboxylic acid (TCA) cycle and oxidative phosphorylation, the production of ATP is increased to yield 30 to 36 molecules [185,186]. Thus, the high metabolism is a result of a significant demand for energy substrate utilization and elevated use of oxygen.

### 4.2. ROS Production and the Ascorbic Acid Metabolic Switch

TCA and oxidative phosphorylation form an efficient mechanism for sustaining synaptic activity. The neuronal glycolytic end-product pyruvate, astrocyte derived lactate, or blood-borne ketonic bodies are great sources to fuel mitochondrial processes [187,188,189]. Neurons change from the use of glucose to lactate through the ascorbic acid metabolic switch, allowing them to bypass glycolysis and oxidize lactate directly in the mitochondria [10,11]. However, the constant use of oxygen by the brain is responsible for the oxidative stress caused by the production of reactive oxygen species [190]. Not only metabolism but also synaptic signaling is a source of oxidative species. High glutamatergic activity causes an increase in extracellular glutamate, activating glutamate receptors (ionotropic NMDA, AMPA, and kainic acid receptors) which produce an elevated uptake of calcium promoting the production of reactive oxygen species [191,192,193,194,195]. Since neurons are highly sensitive to oxidative damage, mechanisms to maintain antioxidant activity are required during physiological activities such as recycling and release of ascorbic acid by astrocytes. However an imbalance in these processes leads to pathological conditions as discussed below.

## 5. Oxidative Stress and Neurodegenerative Disorders

While neurodegenerative diseases present a variety of symptoms and mechanisms which lead to the pathological state, most of them have in common a mitochondrial dysfunction, accumulation of abnormal proteins and production of ROS causing neuronal death. Moreover, increased ROS levels have been observed in post-mortem brain tissue from patients with neurodegenerative disorders, including Amyotrophic lateral sclerosis (ALS), Alzheimer’s disease (AD), Huntington’s disease (HD) and Parkinson’s disease (PD). Overall, the brain tissue is vulnerable to oxidative damage caused by its high oxygen consumption metabolic rate.

### 5.1. Redox Imbalance, Ascorbic Acid and Neurodegeneration

The origin of oxidative stress in neurodegenerative disorders could be related with a redox imbalance, correlating with a deficiency of antioxidant defence enzymes, ascorbic acid supply and an altered ascorbic acid homeostasis in the CNS. Failure to supply or maintain ascorbic acid could affect the oxidative status of the cell. As we mentioned, several neurodegenerative diseases show increased levels of oxidative stress and thus treatment with antioxidant agents, in particular ascorbic acid, has been proposed to be beneficial for patients.

**Figure 2:**
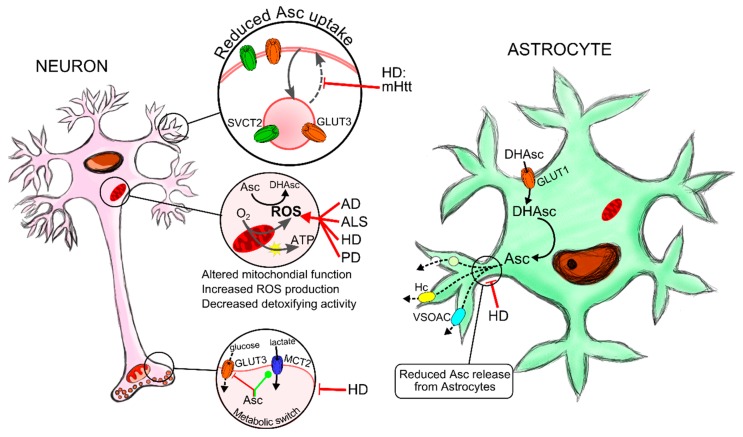
Failure in ascorbic acid homeostasis contributes to neurodegeneration. Neuronal TCA (tricarboxylic acid) and oxidative phosphorylation are highly efficient mechanisms in sustaining synaptic activity. However, the continued use of oxygen generates reactive oxygen species (ROS), which leads to oxidative stress. Therefore, neuronal metabolism and synaptic signalling induce ROS production. Neurons are highly sensitive to oxidative stress and thus ascorbic acid recycling by astrocytes and neuronal uptake through SVCT2 transporters are important mechanisms in maintaining antioxidant defence. During aging as well as in neurodegenerative diseases there is an imbalance in ROS production, decreased levels of antioxidant molecules and impairment in detoxifying enzyme activity such as superoxide dismutase (SOD) or catalase. In HD, accumulation of mutant Huntingtin protein alters mitochondrial biogenesis and expression of antioxidant defence genes, increasing oxidative damage leading to neuronal death. Amyloid β peptide and α-synuclein accumulation in AD and PD respectively, induce ROS production which in turn participates in protein aggregation and neuronal death in both pathologies. SOD1 loss of function due to its mutation is responsible for elevated ROS and causal for a type of ALS. Ascorbic acid levels tend to be reduced in AD, ALS, HD and PD. In HD, we have demonstrated that the failure in astrocytic ascorbic acid release and a decreased neuronal uptake, due to the reduced trafficking of SVCT2 and GLUT3 transporters to the cell surface, are responsible for the impaired metabolic switch, the decreased neuronal antioxidant protection and most likely HD metabolic failure and neuronal death. AD-Alzheimer’s Disease; ALS-Amyotrophic lateral sclerosis; Huntington’s disease-HD; Parkinson’s Disease-PD; mHtt-mutant huntingtin; mSOD-mutant superoxide dismutase; SOD-Superoxide dismutase; ROS-reactive oxygen species.

Here, we show several evidences supporting the involvement of ascorbic acid homeostasis in neurodegenerative diseases (Figure 2).

#### 5.1.1. Amyotrophic Lateral Sclerosis

Amyotrophic lateral sclerosis (ALS) is a chronic neurodegenerative disorder [196]. Damage to motor neurons in the primary motor cortex, corticospinal tracts, brain stem, and spinal cord has been observed, leading to muscle weakness that typifies ALS. The causes of neurodegeneration in ALS are still unknown, but several researches suggest a role for mitochondrial dysfunction, excitotoxicity, cytoskeletal abnormalities, oxidative stress and protein aggregation [197]. Approximately 10% of ALS are familial cases, and 20% are caused by mutations in the superoxide dismutase type 1 (SOD1) gene [198]. Mice expressing mutated SOD1 show calcium impairment, impaired electron transport chain activity and altered ROS production [199,200].

Oxidative stress has been proposed as a key player in the progression of neurodegeneration in ALS. One study suggests systemic oxidative stress in ALS patients, but without significate differences of ascorbic acid plasma levels and CSF levels. Another study showed lower ascorbic acid levels in the CSF from ALS patients in parallel with high levels of ascorbyl radicals, an oxidative stress marker. In ALS transgenic mice, treatment with a high dose of ascorbic acid before onset of the disease shows a beneficial effect, but this does not occur when administered after onset of the disease.

#### 5.1.2. Alzheimer’s Disease

Alzheimer’s Disease (AD) is a highly prevalent neurodegenerative disorder. It is characterized by progressive memory loss and cognitive dysfunction. Patients exhibit neuronal loss at the temporal lobe, parietal lobe, frontal cortex and cingulate gyrus, as well as other subcortical regions [201,202]. A neuropathological change that correlates with AD is the presence of neurofibrillary tangles (aggregates of hyperphosphorylated tau protein) accumulated in the neuronal cytosol and extracellular senile plaques, composed of β-amyloid (Aβ) deposits which are derived from the amyloid precursor protein (APP) [203,204]. Evidence shows that mitochondrial impairment may induce formation of Aβ plaques and neurofibrillary tangles [205,206,207]. Many studies indicate that Aβ increases neuron vulnerability to oxidative stress and impairs the electron transport chain [206]. Oxidative stress induces amyloidogenic APP processing and increases β-secretase activity resulting in an accumulation of Aβ peptide [208,209]. Aβ oligomers interfere with mitochondrial dynamics [210,211]. Several studies demonstrate that Aβ aggregation promotes ROS production [212]. Impairment in synaptic plasticity mediated by Aβ peptide has also been demonstrated [213].

In recent studies, acute parenteral and intravenous treatment with ascorbic acid reversed some of the spatial learning and memory deficits found in AD transgenic mice without changing plaque deposition [214,215,216]. To examine the precise effect of ascorbic acid Kook and colleagues generated a transgenic mouse (5XFAD). These mice have depositions of amyloid plaques in the brain and are unable to synthesize ascorbic acid *in vivo*. They found that treatment with ascorbic acid reduced amyloid plaques, resulting in reduced blood brain barrier disruption and prevention of abnormal mitochondrial morphology. These results suggest that ascorbic acid could provide a protective effect against AD-like pathologies [217]. Ascorbic acid levels in plasma are decreased in AD patients [218,219] and it has been suggested that increased dietary intake may reduce the risk of developing AD [220].

#### 5.1.3. Huntington’s Disease

Huntington’s disease (HD) is a genetic disorder characterized by general neurodegeneration in brain with marked deterioration of medium-sized spiny neurons (MSNs) in the striatum [221]. The disease is caused by a mutation in the gene coding for huntingtin (Htt). Htt is an integrator of intracellular trafficking, favouring transport of vesicles, organelles and proteins to the cell surface [222,223]. Clinical symptoms of HD include a progressive increase in involuntary movements called chorea [224,225], cognitive deterioration [226], dementia and weight loss [227]. Enhanced oxidative damage in HD induces strand breaks in DNA in HD brain [228]. Also, in HD an increase of unsaturated fatty acid peroxidation [229] increased oxidation of proteins and lipids [230], as well an impaired SOD activity [27,231,232] are observed. Defects on brain energy metabolism have been described in presymptomatic and symptomatic subjects [27,233,234,235,236], explained by decreased glucose metabolism, impaired mitochondrial ATP production, calcium homeostasis, trafficking and biogenesis [205,236,237,238,239,240,241,242]. On the other hand, post-mortem analyses in patients shows deficits in the activity of mitochondrial complexes I, II and III [24,238,241,243,244]. 3-Nitropropionic acid (3NP), an inhibitor of the mitochondrial complex II, has been reported to induce selective striatal damage [245]. In cells expressing mutant Htt, 3-NP can induce mitochondrial dysfunction and cell death more easily [246]. Htt aggregates near mitochondria have been observed, suggesting N-terminal interaction with the outer mitochondrial membrane [247,248,249]. Mutant Htt interaction with PGC-1α decreases this factors ability to induce mitochondrial biogenesis [250,251]. PGC-1α impaired function results in increased oxidative damage and neuronal death due down regulation of SOD and GSH peroxidase genes [252].

Impaired ascorbic acid homeostasis is observed in Huntington’s disease animal models [109]. Analysis in animal models expressing mHtt show a decrease in extracellular ascorbic acid levels in the striatum after recovery from anaesthesia [253,254]. Only wild-type mice show an increase in extracellular ascorbic acid after anaesthesia, in contrast, R6/2 mice show the opposite response. A similar response was observed when striatal ascorbic acid release was evoked by cortical stimulation [254]. The loss of extracellular ascorbic acid also impacts striatal neuronal activity [255]. Acuña and colleagues (2013) demonstrated abnormal ascorbic acid release from astrocytes and impaired ascorbic acid uptake in neurons. This should explain alterations in neuronal metabolic substrate preferences in HD. Moreover, in experiments using immortalized striatal neurons expressing mutant huntingtin (STHdhQ cells), the ascorbic acid transporter 2 (SVCT2) fails to translocate to the plasma membrane when extracellular ascorbic acid concentration increases, leading to an impairment of ascorbic acid uptake in HD neurons. We have recently demonstrated that modulation of neuronal metabolism by ascorbic acid is independent of antioxidant properties of this molecule [172]. Thus increased ROS levels observed in HD (and other neurodegenerative diseases) should contribute to metabolic failure through the oxidation of ascorbic acid.

#### 5.1.4. Parkinson’s Disease

Parkinson’s disease (PD) is a common neurodegenerative movement disorder. It is characterized by progressive degeneration of dopaminergic neurons in the substantia nigra pars compacta [256,257], the loss of innervation to the striatum and the subsequent release of dopamine [258,259,260]. Dementia, depression and behavioural deficiencies are also common in the advanced stages of the disease. PD is characterized by Lewy bodies inclusions which correspond to accumulations of α-synuclein in neurons. Mutations in several genes are found in PD, including genes coding for α-synuclein, leucine-rich repeat kinase 2 (LRRK2), DJ-1, PINK1 and ATP13A2 [261].

There are several evidences that mitochondrial dysfunction contributes to the pathogenesis of PD [262]. In fact, mitochondrial dysfunction has been related to mutations in PINK1 and DJ1 genes. Dopamine metabolism produces oxidant species therefore oxidative stress also occurs in PD [263,264,265,266]. On the other hand, oxidative stress participates in protein aggregation in PD. Modifications of the α-synuclein by oxidative stress, including 4-hydroxy-2-nonenal, nitration and oxidation, have been implicated in oligomerization of α-synuclein. These modifications are more likely to form oligomers compared to unmodified α-synuclein [267].

Treatment of PD flies, exhibiting human-α-synuclein in their neurons, with ascorbic acid demonstrated its protective effect as it delayed the loss of climbing ability [268]. Ascorbic acid may prevent cell death of dopaminergic human neurons *in vitro*; this may be due to the inhibition of oxidative stress [269]. Studies of ascorbic acid plasma levels from PD patients compared to controls are contradictory [270,271]. In one study, lymphocyte ascorbic acid levels were lower in PD patients. In the same patient cohort, plasma ascorbic acid levels also tended to be lower. Lymphocyte ascorbic acid levels could act as a potential biomarker for progression of PD [272].

## 6. Conclusions

In summary, ascorbic acid stands out in the defence against oxidative stress in the CNS. Its antioxidant activity is of vital importance, given the elevated ROS production by the high metabolic rate implicated in the specialized and complex functions needed to sustain neurotransmission.

Taking into consideration the different roles of ascorbic acid here described, as an antioxidant molecule, a neuromodulator of synaptic activity and its function on the metabolic switch in neurons during brain activity and resting conditions, this molecule appears as a key factor in physiological and pathological conditions.

Ascorbic acid has been tested as a neuroprotective agent in patients for the treatment of neurodegenerative diseases such as PD and ALS [273]. In the case of HD, favourable data has only been recapitulated from *in vitro* studies [26,143] and pre-clincal tests using mice [253]. While *in vitro* analyzes show very promising results, *in vivo* assays show modest improvements of the symptomatology. One of the major problems in the use of ascorbic acid in neuroprotective therapy is associated with the low stability of this molecule. Ascorbic acid is rapidly oxidized in solutions and when in contact with the air.

Neurodegenerative diseases which exhibit high oxidative stress constantly consume ascorbic acid available in the brain [172]. When ascorbic acid is used as an antioxidant, it is oxidized. Therefore in the presence of high levels of ROS no ascorbic acid remains available to modulate neuronal metabolism. Thus oxidative stress, elevated ROS production and the failure of homeostatic systems for ascorbic acid recycling are crucial aspects in the progression of neurodegeneration. Therefore, it is important to continue the efforts to thoroughly evaluate the roles of ascorbic acid for the development of new therapies against neurodegenerative disorders.

## Abbreviations

Aβ: β-amyloid peptide; AD: Alzheimer’s disease; ALS: Amyotrophic lateral sclerosis; AMPAR: α-amino-3-hydroxy-5-methyl-4-isoxazolepropionic acid receptor; ANLS: astrocyte-neuron lactate shuttle; ATP: adenosine triphosphat; APP: amyloid precursor protein; BBB: blood-brain barrier; Ca^+2^ (v) 3.2 T-channel: neuronal t-type calcium channel; CSF: cerebrospinal fluid barrier; CNS: Central Nervous System; GABA: γ amino butyric acid; GLT1: astrocytic glutamate transporter; GLAST: astrocytic glutamate transporter; GLUTs: glucose transporters; GSH: glutathione, c-l-glutamyl-l-cysteinylglycine; HD: Huntington’s disease; HTT: huntingtin protein; OH8dG: 8-hydroxy-2′-deoxyguanosine; LRRK2: leucine-rich repeat kinase 2; LTP: long-term potentiation process; MCTs: monocarboxylate transporter; MSSNs: medium-sized spiny neurons; NADPH: Nicotinamide adenine dinucleotide phosphate; 3NP: 3-Nitropropionic acid; NMDAR: *N*-methyl-d-aspartate receptor; PD: Parkinson’s disease; PGC-1α: proliferator-activated receptor γ co-activator 1α; ROS: reactive oxygen species; SLC2: family of glucose transporters; SLC23: family of ascorbate transporters; SOD1: superoxide dismutase 1; SVCTs: Sodium dependent vitamin C transporter; TCA: tricarboxylic acid; VSOAC: volume-sensitive organic osmolyte-anion channel.
